# Can pluripotent/multipotent stem cells reverse Parkinson’s disease progression?

**DOI:** 10.3389/fnins.2024.1210447

**Published:** 2024-01-31

**Authors:** Yongkang Wu, Xiangtian Meng, Wai-Yin Cheng, Zhichao Yan, Keqin Li, Jian Wang, Tianfang Jiang, Fei Zhou, Ka-Hing Wong, Chunlong Zhong, Yi Dong, Shane Gao

**Affiliations:** ^1^Key Laboratory of Adolescent Health Evaluation and Sports Intervention, Ministry of Education, East China Normal University, Shanghai, China; ^2^Department of Neurosurgery, Shanghai East Hospital, School of Medicine, Tongji University, Shanghai, China; ^3^Research Institute for Future Food, The Hong Kong Polytechnic University, Hong Kong, Hong Kong SAR, China; ^4^Department of Food Science and Nutrition, The Hong Kong Polytechnic University, Hong Kong, Hong Kong SAR, China; ^5^Department of Neurology, Shanghai Eighth People’s Hospital Affiliated to Jiangsu University, Shanghai, China; ^6^Department of Neurology, Third Affiliated Hospital of Navy Military Medical University, Shanghai, China

**Keywords:** pluripotent stem cells, multipotent stem cells, Parkinson’s disease, dopamine, transplantation

## Abstract

Parkinson’s disease (PD) is a progressive neurodegenerative disorder characterized by continuous and selective degeneration or death of dopamine neurons in the midbrain, leading to dysfunction of the nigrostriatal neural circuits. Current clinical treatments for PD include drug treatment and surgery, which provide short-term relief of symptoms but are associated with many side effects and cannot reverse the progression of PD. Pluripotent/multipotent stem cells possess a self-renewal capacity and the potential to differentiate into dopaminergic neurons. Transplantation of pluripotent/multipotent stem cells or dopaminergic neurons derived from these cells is a promising strategy for the complete repair of damaged neural circuits in PD. This article reviews and summarizes the current preclinical/clinical treatments for PD, their efficacies, and the advantages/disadvantages of various stem cells, including pluripotent and multipotent stem cells, to provide a detailed overview of how these cells can be applied in the treatment of PD, as well as the challenges and bottlenecks that need to be overcome in future translational studies.

## 1 Introduction

Parkinson’s disease (PD), a common chronic neurodegenerative disease that is second only to Alzheimer’s disease in incidence, is one of the most common movement disorders and is sometimes accompanied by emotional disturbances. Its clinical manifestations include slow movement, muscle stiffness, resting tremor, and postural instability (Obeso et al., 2022). PD affects approximately 0.3% of the population, with the proportion reaching 1.0% among individuals over 60 years old. In the United States alone, more than 200,000 people suffer from PD (Cui et al., 2020). The main pathological feature of this disease, which can be sporadic or familial, is the degeneration of dopamine (DA) neurons in the substantia nigra pars compacta (SNpc), which leads to the loss of axonal projections and decreased release of DA into the striatum. Despite extensive research on the roles of genetics, the environment, and aging in PD pathogenesis, the underlying cause of PD has yet to be clarified, making the diagnosis and prognosis of the disease challenging. Currently, the key clinical treatments for PD are medicinal and surgical interventions, but these treatments only alleviate symptoms and cannot prevent neurodegeneration or reverse the loss of midbrain dopamine (mDA) neurons; moreover, they cannot improve the quality of life of patients (Mollenhauer and von Arnim, 2022; Obeso et al., 2022; The Lancet and Neurology, 2022). Therefore, scientists are attempting to treat PD through stem cell transplantation. Stem cell therapy involves transplanting seeding cells to the lesion or another specific site through an optimized delivery route to achieve tissue regeneration and repair and thus cure specific diseases (Barbuti et al., 2021). The potential of stem cell transplantation in PD treatment was first demonstrated in the 1980s through a study in PD patients who underwent foetal brain tissue transplantation (Shariati et al., 2020). In recent years, scientists have been developing safe and convenient stem cell therapies with better transplantation efficiency and treatment efficacy and reduced side effects by improving culturing techniques. Among the many stem cell transplantation strategies, stem cell-derived dopamine (SC-DA) neuron transplantation is considered the most promising for treating PD, as it involves transplanting DA neurons into the SNpc to replace lost neurons and restore DA neurotransmission in PD patients (Sonntag et al., 2018; Parmar et al., 2020). Currently, there is a diverse range of stem cell sources for PD treatment, and different types of stem cells have shown potential advantages and disadvantages in PD treatment. This review summarizes preclinical and clinical data for various types of stem cells in the treatment of PD and provides a detailed analysis of the existing data, recent advancements, and research directions on the use of human pluripotent/multipotent stem cells for treating PD, providing a foundation for the development of stem cell therapies for PD.

## 2 Mechanisms of Parkinson’s disease

**FIGURE 1:**
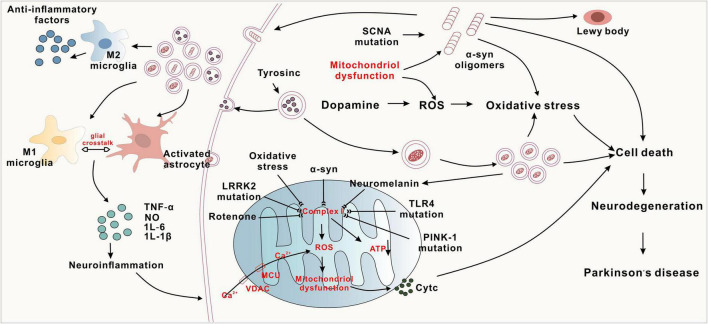
The complex pathogenesis of Parkinson’s disease.

The aetiology and pathogenesis of PD involve abnormal aggregation of α-synuclein (α-syn), mitochondrial dysfunction, oxidative stress, and neuroinflammation (Figure 1).

### 2.1 Abnormal aggregation of alpha-synuclein

α-Syn is an intracellular neuronal protein consisting of 140 amino acid residues that is located mainly at the presynaptic terminal; this synaptic terminal mediates neurotransmitter release and can lead to neuronal damage, resulting in neurodegeneration of the central nervous system. α-Syn is found in the cerebral cortex of PD patients and is highly expressed in the hippocampus and olfactory bulb (Obeso et al., 2022). α-Syn is a major component of Lewy bodies (LBs) (Choong and Mochizuki, 2022), which are a typical pathological feature of PD. When α-syn misfolds and aggregates abnormally under the influence of environmental toxins, bacterial infections, and viruses, dopaminergic neurons become progressively unhealthy and undergo apoptosis, leading to PD (Tofaris, 2022; Vidović and Rikalovic, 2022). Increased α-syn and poor degradation of cytosolic proteins are the main causes of aggregation. A novel peptide mimetic, FN075, was found to promote endogenous α-syn aggregation *in vivo*, and injection of FN075 into the substantia nigra of rats resulted in motor dysfunction and loss of dopaminergic neurons, suggesting that α-syn aggregation is associated with the development of PD (Olsen et al., 2019). Disruption of the glial cell function of removing extracellularly aggregated α-syn through autophagy also leads to degeneration of dopaminergic neurons (Nash et al., 2017; Choi et al., 2020). Knockdown of an oxidative stress sensor (DJ-1) in microglia resulted in an increase in its neurotoxicity to dopaminergic neurons and an inflammatory response to α-syn, which impaired the cellular uptake and clearance of α-syn (Nash et al., 2017). Hua et al. (2019) found that astrocytes capture α-syn through endocytosis and transport it to lysosomes to play a scavenging role, and if the capture capacity is greater than the degradation capacity for a prolonged period of time, α-syn accumulates in astrocytes, leading to the development of PD. This recent study suggests that misfolded α-syn may have a prion-like mode of action and diffusion properties, allowing it to be transmitted across synapses and transported to other brain regions via axons, which leads to the production of more α-syn oligomers in the brain, causing degenerative death of dopaminergic neurons and manifesting clinical symptoms of PD (Ma et al., 2019). In addition, the aggregates formed by α-syn misfolding can propagate between neighboring neuronal cells under the action of lymphocyte activation gene 3 (LAG3), accelerating neuronal cell damage and leading to the onset and progressive development of PD (Zhang et al., 2021). Aberrant α-syn aggregation plays an important role in various mechanisms of PD pathogenesis, causing oxidative stress, mitochondrial dysfunction, neuroinflammation, and axonal damage. Therefore, preventing the abnormal aggregation of α-syn has become one of the core issues in related research.

### 2.2 Neuroinflammation

Neuroinflammation is another danger of PD, caused mainly by the overreaction of microglia and astrocytes, which release several inflammatory factors. In addition, there is a vicious cycle between neuroinflammation and α-synuclein aggregation, which exacerbates the degeneration of dopaminergic neurons (Wang et al., 2023). Overactive microglia release several proinflammatory cytokines (IL-1β, IL-6, TNF-α) and chemokines that accelerate neuroinflammation. In the MPTP-induced mouse PD model, inhibition of microglia activation reduces TNF-α secretion and prevents the loss of DA neurons (He et al., 2017). Glial cell maturation factor (GMF) is a recently discovered proinflammatory protein, and overexpression of GMF in glial cells leads to neuroinflammation and neurodegeneration (Ahmed et al., 2020; Wang et al., 2023). Knockdown of GMF in BV2 microglia by CRISPR/Cas9 targeting inhibited microglia activation, which resulted in a reduction of the inflammatory burden and an improvement in motor performance (Selvakumar et al., 2019, 2020). This suggests that that GMF-targeting drugs may be useful for future therapeutic intervention in PD. Astrocytes are more bulky neuronal support cells. Activation of the nuclear factor kB (NF-kB) signaling pathway in astrocytes releases large amounts of inflammatory factors such as TNF-α and IL-1β, which aggravate neuronal damage (Sun et al., 2016). These results suggest that glial cells play a key role in the pathogenesis of PD, and in particular, the release of inflammatory cytokines in dopaminergic neurons may be one of the main mechanisms responsible for PD pathogenesis. In addition, inflammatory responses mediated by peripheral immune cells may be involved in the development of PD. Activated microglia and peripheral immune cells infiltrate the substantia nigra and striatum in both PD patients and animal models of PD. In summary, immune and inflammatory responses accompany the onset and progression of PD, but the exact mechanisms are unclear. All these findings suggest that glial cell-induced neuroinflammation plays a key role in the pathogenesis of PD.

### 2.3 Oxidative stress

Reactive oxygen species (ROS) are a class of highly biologically active oxygen-containing molecules. Under normal physiological conditions, ROS can regulate various signaling pathways involved in redox reactions and play an important defensive role in the organism, but the overproduction of ROS leads to oxidative stress, which is the main driver of the pathogenesis of PD (Singh et al., 2019). In PD patients, ROS produced under oxidative stress cause the energy demand of nigrostriatal dopaminergic neurons to exceed energy In Parkinson’s disease patients, ROS generated under oxidative stress cause the energy demand of nigrostriatal dopaminergic neurons to exceed their energy supply, ultimately leading to DA neuronal damage and death (Trist et al., 2019). In addition, ROS also lead to mitochondrial autophagy impairment, biomolecule damage, and microglial overreactivity, as well as promoting the release of inflammatory factors and inducing neuroinflammation (Ding et al., 2018; Imbriani et al., 2022). Diethyl ketone has been found to lead to the aggregation of α-syn in the nigra in the mouse brain via the oxidative stress pathway, which leads to the damage of DA neurons. Nrf2 is a transcription factor involved in the regulation of the cellular redox response. Nrf2 can regulate antioxidant enzymes and gene expression through mediation of the redox pathway and can increase cellular resistance to ROS (Yang et al., 2022). In addition, Toll-like receptor 4 (TLR4) plays a major role in the regulation of activator protein-1 (AP-1), a redox-sensitive transcription factor that mediates the response of numerous genes to a wide range of physiological and pathological stimuli, including ROS. Studies have reported decreased AP-1 expression in dopaminergic neurons, less reduction in ROS content, and improved dyskinesia in PD mice in the MPTP model of TLR4-knockout (KO) mice, suggesting that TLR4 plays a key role in the pathogenesis of PD (Campolo et al., 2019; Heidari et al., 2022). Oxidative stress is a common central nervous system (CNS) response caused by multiple pathogenic mechanisms that affect the prognosis of PD and is usually not the first manifestation of PD.

### 2.4 Mitochondrial dysfunction

Mitochondrial dysfunction damages neurons and glial cells, leading to decreased adenosine triphosphate (ATP), increased free radicals, imbalance in calcium homeostasis, mutations in mitochondrial DNA (mtDNA), and production of abnormal proteins that dock with mitochondria through multiple pathways, which in turn cause neurodegeneration in PD. Peroxisome proliferator-activated receptor-γ coactivator-1α (PGC-1α) is a coactivator of several transcription factors and a major regulator of mitochondrial biogenesis. In cellular models, PGC-1α upregulates the nuclear coding subunit of the mitochondrial respiratory chain and prevents dopaminergic neuron loss and neurotoxicity induced by rotenone or mutant α-syn (Zheng et al., 2010). IL-6 is a proinflammatory cytokine, and impaired mitochondrial autophagy and subsequent mtDNA release leads to elevated IL-6 levels. It has been shown that about 20 PD susceptibility genes (PRKN, *SNCA*, etc.) are involved in the process of mitochondrial autophagy and regulation of mitochondrial function, and patients with inherited PD caused by mutations in these genes present with impairment of the mitochondrial autophagic pathway and mitochondrial dysfunction. PRKN deficiency causes impaired mitochondrial autophagy leading to the release of mtDNA, which triggers inflammation; Borsche et al. (2020) found that the clinical features of PD associated with PRKN/PINK1 double allele mutations were an earlier age of onset, longer duration of disease, and reduced olfactory impairment. PINK1 is particularly prominent in mitochondrial autophagy, and mutations in PINK1 disrupt several parts of mitochondrial biology, resulting in mitochondrial dysfunction and leading to ineffective clearance of damaged mitochondria, which in turn triggers inflammation and the death of dopaminergic neurons, ultimately resulting in the development of PD (Ando et al., 2017). In addition, LRRK2 mutations can affect mitochondrial axonal transport, which in turn affects mitochondrial function and plays an important role in the pathogenesis of PD, but LRRK2 kinase inhibitors have not yet been able to rescue this defect (Hsieh et al., 2016; Jeong and Lee, 2020). All these studies suggest that systemic mitochondrial dysfunction triggers neurodegeneration in the substantia nigra striata.

Research on the mechanism of PD is extensive and has made great progress, but there is a lack of relevant clinical research. Current PD therapeutic measures still cannot completely cure PD symptoms, and the development of PD therapeutic drugs needs to focus more on multipathway and multitarget research, such as screening for compounds that can inhibit oxidative stress as well as protect mitochondrial function. Therefore, it is important to explore the pathogenesis of PD and prevent the degeneration of nigrostriatal dopaminergic neurons.

(1) α-synuclein aggregation, genetic mutations, oxidative stress, and neuroinflammation inhibit complex-I, leading to reduced ATP synthesis and mitochondrial dysfunction. Subsequently, Cyt C activation leads to cell death. (2) The conversion of tyrosine to dopamine and the abnormal increase in dopamine metabolism promote oxidative stress and the accumulation of neuromelanin. (3) Activation of both M1 and M2 microglial cells, along with astrocyte activation, leads to the release of inflammatory factors such as TNF-α and IL-6, resulting in neuronal death. Conversely, M2 microglia release anti-inflammatory factors, exerting neuroprotective effects.

## 3 Current common treatments for PD

Clinical interventions for PD primarily consist of pharmacological treatment with levodopa (L-DOPA) combined with lifestyle modifications such as dietary adjustments and exercise, as well as deep brain stimulation (DBS) therapy. However, these treatments are unable to reverse the progression of PD. As the disease progresses, PD patients experience a significant decline in quality of life, resulting in substantial economic and emotional burdens for both families and society (Mak et al., 2017; Krauss et al., 2021; Vijiaratnam et al., 2021).

### 3.1 Pharmacological treatment

The dopaminergic system is the primary therapeutic target for the clinical management of PD. Promoting mDA neurotransmission, stimulating central dopamine receptors, and inhibiting the loss of central dopamine transmission can somewhat alleviate the adverse effects of continuous dopamine neuron degeneration. According to their targets and effects, dopaminergic drugs can be classified as dopaminergic precursors, dopamine receptor agonists (DAs), selective monoamine oxidase-B inhibitors (MAO-BIs), and catechol-*O*-methyltransferase inhibitors (COMTIs). Other PD drugs include anticholinergics and amantadine. L-DOPA is the precursor of dopamine; it can cross the blood-brain barrier (BBB) and be converted to dopamine by DOPA decarboxylase in the CNS, where it promotes midbrain dopamine neurotransmission and thus alleviates PD symptoms. L-DOPA is the most common drug used for the treatment of PD (Fox, 2022). To prevent L-DOPA from diffusing into the periphery before decarboxylation and causing adverse effects, peripheral DOPA decarboxylase inhibitors are usually used in combination with L-DOPA. Peripheral DOPA decarboxylase inhibitors exert their effects by increasing circulating L-DOPA levels while reducing CNS L-DOPA levels (Beckers et al., 2022). However, systemic administration of L-DOPA may cause off-target effects (such as nausea, somnolence, and orthostatic hypotension). For advanced PD, levodopa-carbidopa intestinal gel (LCIG) can be delivered via percutaneous endoscopic gastrostomy into the duodenum or upper jejunum to maintain stable drug concentrations in the blood and reduce phase stimulation of the dopaminergic system by the drug (Fernandez et al., 2015). Pharmacotherapy is a convenient treatment strategy for PD patients, and clinical data have demonstrated its safety, tolerability, and efficacy. However, disease progression and long-term high-frequency drug use make patients susceptible to motor complications and end-of-dose phenomena (Antonini et al., 2011).

### 3.2 Surgical treatment

In cases when pharmacological interventions fail to control the symptoms of PD, surgical interventions may represent alternative strategies for improving prognosis. DBS involves the implantation of electrodes in specific regions of the brain responsible for motor control to manipulate abnormal neural circuitry and thus alleviate or control PD symptoms. Introduced in the 1970s, this treatment involves implanting stimulating electrodes in brain nuclei or specific brain regions and modulating the function of the relevant nuclei or regions through pulsed electrical stimulation to improve symptoms. Although the exact mechanism of action is not fully understood, evidence suggests that DBS modulates the signals of abnormal neurotransmission pathways by stimulating nodes in complex neural networks. The common targets of DBS in the treatment of PD are the globus pallidus medialis (GPi) and the thalamic nucleus (STN). Both targets are nodes in the frontal-basal ganglia-thalamo-cortical network, which is involved in the regulation of cognitive, behavioral, and motor functions. Abnormal signaling within this network is associated with motor and non-motor symptoms of PD; the STN is part of indirect and direct pathways, while the GPi is the principal efferent nucleus of the basal ganglia system. Several clinical studies have shown that bilateral DBS of both targets improves PD symptoms, but postoperative stimulation-induced adverse effects, as well as psychiatric and cognitive effects, are more common with STN DBS; other adverse effects include dysphagia, speech abnormalities, and gait and balance dysfunction. STN DBS offers improved regulation of anomalous movements and an amplified capability to adjust medication. STN DBS has the potential to reduce medication usage (Krauss et al., 2021; Baumgartner et al., 2022; Flouty et al., 2022). Some studies indicate that approximately 50% of patients experience significant symptom improvement and reduced drug dosages following surgery. However, it is necessary for patients to continue taking their medication after surgery in conjunction with the surgical procedure to attain a favorable therapeutic outcome (Limousin and Foltynie, 2019). A large quantity of clinical evidence has shown that DBS can effectively alleviate tremors, bradykinesia, and muscle rigidity, thus improving patients’ quality of life. However, DBS has significant limitations, including a high cost; risks associated with electrode implantation, such as permanent cognitive impairment; and the need for electrode replacement due to damage, erosion, or migration over time (Krauss et al., 2021; Reich et al., 2022).

### 3.3 Gene therapy

In recent years, gene therapy has gained increasing attention from scholars both at home and abroad. The main strategy of gene therapy for PD is using plasmid vectors or the CRISPR/Cas9 system to edit disease-related mutant genes, such as Parkinson disease protein 2 (PARK2), Parkinson disease protein 7 (PARK7), and leucine-rich repeat kinase 2 (LRRK2), to alter their transcription or translation and thus promote the release of DA and glial cell-derived neurotrophic factor (GDNF), ultimately increasing DA levels and restoring the function of midbrain neural circuitry. The most straightforward approach to enhance DA production in the nucleus accumbens is increasing the enzyme expression in the DA pathway. This includes the DA rate-limiting enzymes TH and AADC, and the TH cofactor tetrahydrobiopterin rate-limiting enzyme GCH1. A non-human primate model was utilized to examine the changes in striatal AADC expression regulated by viral transduction. Positron emission tomography (PET) demonstrated increased AADC activity and enhanced motor function, providing confirmation of the efficacy of gene therapy for PD (Axelsen and Woldbye, 2018; Merola et al., 2020). Compared with the administration of DA-related drugs, gene therapy is more precise and can provide more sustained therapeutic effects. However, gene therapy may pose some potential risks, such as immune reactions and off-target effects caused by the introduced gene mutations (Axelsen and Woldbye, 2018; Niethammer et al., 2018).

## 4 Stem cell transplantation for PD

### 4.1 Stem cell therapy for PD

**FIGURE 2:**
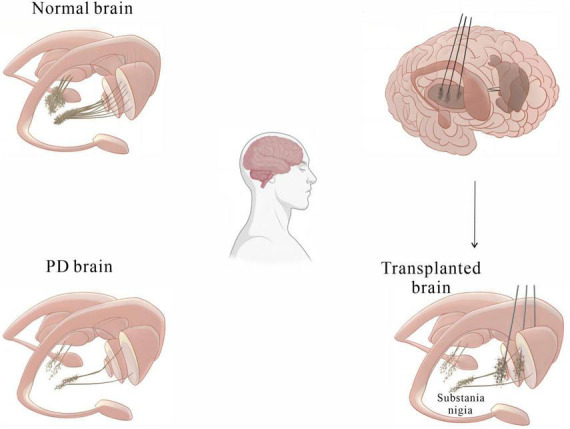
A basic diagram showing how cell transplantation therapy can alleviate motor symptoms in PD patients.

Perlow et al. (1979) made a ground-breaking discovery, finding that transplanting foetal midbrain tissue containing DA-producing neurons into the substantia nigra of PD rats improved motor function. Further preclinical studies have demonstrated that transplantation of midbrain tissue into PD models lacking nigrostriatal projections resulted in the formation of synapses with host striatal neurons and successful integration of these neurons into host neural circuits, reversing the behavioral symptoms of PD (Björklund et al., 1980, 1981; Dunnett et al., 1981). These findings provide a theoretical basis for studying cell transplantation strategies in clinical trials. Kordower et al. (1995) transplanted midbrain tissue derived from human embryos into the putamen of PD patients. One year post-surgery, it was found that the grafts had not only survived but also resulted in obvious dopaminergic reinnervation of the striatum. Improved function of the neurons surrounding the grafts was observed by PET scans (Kordower et al., 1995). Li et al. (2016) studied a patient who had received foetal stem cell transplants 24 years prior and found over 40,000 surviving DA neurons in the putamen, with their axons extending into the host striatum to form neural circuits. In clinical trials, aggregated α-syn, a hallmark of PD pathology, was found in dopaminergic neurons in grafted tissue in some PD patients (Kordower et al., 2008; Barker et al., 2013; Li et al., 2016), while no α-syn aggregates were detected in other patients, indicating that the presence of pathological changes in the foetal tissue graft depends on the individual patient (Kriks et al., 2011; Politis et al., 2011; Hallett et al., 2014). These findings indicate that human foetal midbrain tissue transplantation is a potential treatment for PD (Figure 2). However, the clinical application of this therapy is hindered by several major issues. First, obtaining human foetal tissue and extracting DA-producing neurons from the ventral midbrain remains challenging. Second, the adverse reactions caused by foetal midbrain tissue implantation remain unclear. Finally, the ethical and moral issues of applying deceased aborted foetal tissue to treat PD patients need to be addressed. Nonetheless, the clinical studies referenced above confirm that cell transplantation is one of the most promising therapies for PD for the following reasons:

? Selective loss of mDA neurons is the most prominent pathological feature of PD.

? Transplanted cells can reinnervate the striatum, restore DA neural circuitry function, and ameliorate PD-related motor deficits for a sustained period in some patients.

### 4.2 mDA neuron development and maintenance

The *in vitro* induction of cell differentiation into mature mDA neurons goes through multiple stages. (1) Pluripotent stem cell stage: At this stage, stem cells have the potential for self-renewal, replication and multidirectional differentiation and can proliferate in the stem cell maintenance medium while maintaining their multidirectional differentiation potential. (2) Neural precursor cell stage: For pluripotent stem cells to differentiate into various types of neurons, they first need to differentiate into neural precursor cells. In N2B27 neural differentiation medium, the neural precursor cells can differentiate into different neural tissues and highly express neural precursor cell markers such as Pax6, stem cell transcription factor 2 (Sox2), Nestin, Sox1, etc. (Mendes-Pinheiro et al., 2018). Early in the cell culture process, Wnt1 and FOXA play crucial roles in the generation of mDA neurons. Early neural development involves the formation of the isthmic organizer (IsO), which determines the fate of the midbrain (Arenas et al., 2015). The transcription factors Otx2 and Gbx2 induce the formation of the IsO and the midbrain-hindbrain boundary (MHB) (Sunmonu et al., 2011). Otx2 regulates the expression of Wnt1 in the midbrain (Joyner et al., 2000), while a mutual feedback mechanism between Wnt1 and FGF8 promotes the proper positioning of the MHB. Wnt1 can also promote the formation of mDA neurons by activating the Otx2-Wnt1-LMX1A/Msx1 pathway. Shh-FOXA2 signaling is the second major signaling event involved in midbrain formation. When Shh binds to the transmembrane protein Patched, it promotes the accumulation of the smoothened protein on the cell surface, thus upregulating the expression of ventral transcription factors (Carballo et al., 2018). FOXA2 can inhibit the activity of Hedgehog family proteins, which drive cells to adopt a dorsal midbrain fate (Norton et al., 2005). FOXA1 also inhibits the development of cells into serotoninergic neurons through Nkx38.39 (Volpicelli et al., 2020). After cells differentiate into mDA neurons, transforming growth factor-β3 (TGF-β3), GDNF, brain-derived neurotrophic factor (BDNF), and other factors maintain their survival (Yeap et al., 2023).

### 4.3 Application of pluripotent/multipotent stem cells in the clinical treatment of PD

Stem cells are a class of non-specific cells with self-renewal and multilineage differentiation potential, and they have the potential to induce tissue regeneration to combat degenerative diseases. In clinical treatment, isolated and cultured stem cells are injected into sites of tissue damage, where they may replace damaged cells or promote endogenous cells to regenerate through paracrine effects (Table 1). As human embryos develop, the differentiation potential of stem cells gradually decreases. According to their differentiation potential, stem cells can be classified as totipotent, pluripotent, multipotent, and unipotent stem cells (Jin, 2017; Yamanaka, 2020). In recent years, significant progress in cell culture technologies has enabled researchers to generate many DA neurons from embryonic stem cells (ESCs), induced pluripotent stem cells (iPSCs) and mesenchymal stem cells (MSCs). This has dramatically renewed our hope for the clinical translation of cell therapy for PD. However, due to their differing characteristics, stem cells exhibit their own advantages and disadvantages as seeding cells in the treatment of PD. In this review, we discuss the application of human pluripotent/multipotent stem cells in PD treatment, the bottleneck in the application of these cells, the mechanism of these cells and recent advances in related research, especially clinical trials (Table 2).

**TABLE 1:** Current clinical trials on pluripotent/multipotent stem cell therapy for Parkinson’s disease.

Source of PSCs	Title	Sponsor	Status	Number of cells	Therapy method	Clinical trial number
ESCs	Stereotaxic Transplantation of hAESCs for Parkinson’s Disease	Shanghai East Hospital	Active, not recruiting	50 million hAESCs into the lateral ventricle	Stereoscopic injection	NCT04414813
ESCs	A Trial to Determine the Safety and Tolerability of Transplanted Stem Cell Derived Dopamine Neurons to the Brains of Individuals with Parkinson’s Disease	Region Skane	Recruiting	3.54 million STEM-PD cells per putamen	Stereoscopic injection	NCT05635409
ESCs	Safety and Tolerability Study of MSK-DA01 Cell Therapy for Advanced Parkinson’s Disease	University of California Irvine, Orange	Active, not recruiting			NCT04802733
ESCs	The Precise Transplantation of Human Amniotic Epithelial Stem Cells Into the Ventricle Through Surgical Robot in the Treatment of Parkinson’s Disease.	Shanghai East Hospital	Recruiting		Injection	NCT05435755
ESCs	A Single Center, Open, Single Dosing, Dose-escalation, Phase 1/2a Study to Evaluate the Safety and Exploratory Efficacy of Embryonic Stem Cell-derived A9 Dopamine Progenitor Cell (A9-DPC) Therapy in Patients With Parkinson’s Disease	S.Biomedics Co., Ltd	Recruiting			NCT05887466
iPSCs	Long-term Follow-Up Study after Transplantation of Human iPSC-Derived Dopaminergic Progenitors in the Treatment of Parkinson’s Disease	Kyoto University Hospital	Recruiting			UMIN000048476
iPSCs	Safety and Efficacy of Autologous Induced Neural Stem Cell-derived Dopaminergic Precursor Cells in the Treatment of Parkinson’s Disease	Xuanwu Hospital, Beijing	Recruiting		Injection	NCT05901818
iPSCs	A Clinical Trial of Parkinson’s Disease Treatment by Human-induced Pluripotent Stem Cells (hiPSCs) Derived Dopaminergic Neural Precursor Cells	Shanghai East Hospital	Recruiting		Injection	NCT06145711
BM-MSCs	Phase IIa Randomized Placebo Controlled Trial: Mesenchymal Stem Cells as a Disease-modifying Therapy for iPD	The University of Texas Health Science Center, Houston	Active, not recruiting	10^6 MSCs/kg per treatment	Injection	NCT04506073
BM-MSCs	*Clinical Study on the Efficacy of Natural Stem Cell Mobilizers on Parkinson Disease*	Sociedad Española de Medicina Regenerativa y Terapia Celular	Recruiting		Dietary Supplement	NCT05699694
BM-MSCs	Neurologic Bone Marrow Derived Stem Cell Treatment Study	MD Stem Cells	Recruiting		Intravenous and Intranasal	NCT02795052
AD-MSCs	Single Intradetrusor Injection Of Autologous Adipose-derived Stem Cells In Parkinson’s Disease Patients With Overactive Bladder: A Pilot Study	Al Zahra Hospital			Infusion	UMIN000044106
AD-MSCs	Evaluating Safety, Tolerability, and Efficacy of Autologous MitoCell Transplantation in Subjects with Idiopathic Parkinson’s Disease	Taiwan Mitochondrion Applied Technology Co., Ltd.	Not yet recruiting	3 × 10^7^ cells per hemisphere	Stereoscopic injection	NCT05094011
	*Randomized Clinical Trial in Parkinson’s Disease Patients Using Pluripotent Adipose Stem Cells (PASCs)*	ClusterXStem-Costa Rica	Active, not recruiting		Infusion	NCT06141317
	*Intermediate Size Expanded Access Protocol Using Autologous HB-adMSCs for the Treatment of Parkinson’s Disease*	Hope Biosciences Stem Cell Research Foundation			Infusion	NCT06056427
AD-MSCs	Hope Biosciences Stem Cell Research Foundation	Hope Biosciences Stem Cell Research Foundation	Recruiting			NCT04995081
UC-MSCs	Use of Mesenchymal Stem Cells (MSCs) Differentiated Into Neural Stem Cells (NSCs) in People With Parkinson’s (PD).	University of Jordan	Active, not recruiting			NCT03684122
UC-MSCs	Evaluation of the therapeutic effect of umbilical cord-derived mesenchymal stem cells transplantation on Parkinson’s disease	Xiang’an Hospital Affiliated to Xiamen University	Active, not recruiting			ChiCTR2100043584
UC-MSCs	Safety of Cultured Allogeneic Adult Umbilical Cord Derived Mesenchymal Stem Cells for Parkinson’s Disease	The Foundation for Orthopedics and Regenerative Medicine	Not yet recruiting	Single administration of 100 million cells	Injection	NCT05152394

1. https://clinicaltrials.gov

2. https://trialsearch.who.int

**TABLE 2:** Advantages and disadvantages of pluripotent/multipotent stem cell therapy for PD.

Cell type	Advantages	Disadvantages
ESCs	Has a wide differentiation potential and can differentiate into almost any type of cell	Moral and ethical issues; the use of embryos can be controversial
	Can produce many cells for transplantation or research	Higher risk of immune rejection and need for immunosuppression
	Can effectively replace damaged dopamine neurons, thereby exerting a stronger therapeutic effect than other cell types	Precise differentiation and culture conditions are needed, and control of cell purity and consistency is difficult
iPSCs	Wide range of sources to avoid ethical issues	Low reprogramming efficiency and number of induced multifunctional stem cells generated
	Can be obtained from the patient’s own cells, reducing the risk of immune rejection	Further research is needed to ensure their safety and long-term effectiveness
	Can be used for individualized treatment strategies	
MSCs	Wide range of sources; can be obtained from adult adipose tissue, bone marrow, etc.	Poor cell survival after transplantation; need to improve transplantation methods and techniques
	Low risk of immune rejection for allogeneic transplantation	Challenges related to production and process standardization such as issues with cell purity, batch consistency, etc.
	Have anti-inflammatory properties and tissue repair capabilities and may exert adjuvant therapeutic effects through mechanisms such as promoting cell survival and improving the local environment	Further research is still needed to determine their safety and long-term effects

#### 4.3.1 Clinical applications of embryonic stem cells in treating PD

Embryonic stem cells are a type of clonal cell derived from the inner cell mass of blastocysts; they are pluripotent and thus have the ability to grow continuously in an undifferentiated state and to differentiate into nearly all cell types in the body upon induction (Lindvall, 2016). They are a good cell source of dopaminergic neurons for transplantation into the SNpc and striatum of patients with PD. Initially, ESCs were obtained from aborted foetuses or *in vitro*-fertilized embryos, which raised ethical concerns. Currently, there are multiple methods for obtaining ESCs, such as activating unfertilized oocytes to develop into parthenogenetic embryos, injecting sperm into enucleated oocytes to generate androgenetic embryos, and transferring somatic cell nuclei into enucleated oocytes to induce reprogramming and establish ESC lines (Leeb and Wutz, 2011; Elling et al., 2019). A Swedish survey reported that over half of the population had a positive attitude toward using ESCs in PD drug development (Grauman et al., 2022). Studies have shown that early ESCs can differentiate into DA neurons and improve behavior when transplanted directly into the brains of rodents (Kim et al., 2002; Ben-Hur et al., 2004), but there is a risk of teratoma formation due to the presence of undifferentiated neural epithelial cells among the transplanted cells (Roy et al., 2006); therefore, complete induction of ESC differentiation is needed before transplantation. To overcome this limitation, Kriks et al. (2011) induced the differentiation of ESCs into dopaminergic neuron progenitor cells by dual inhibition of SMAD and the hedgehog gene, and these dopaminergic neuron progenitor cells differentiated into melanin-secreting dopaminergic neurons in the striatum of PD animal models. The seeding cells survived well in the striatum of PD model mice for a relatively long time (over 4 months); functioned like normal dopaminergic cells, as determined by patch–clamp recordings; fully rescued rotation behavior; and partially improved forelimb function (Kriks et al., 2011). Adler et al. (2020) employed a rabies virus tracing technique to demonstrate that dopaminergic neurons implanted in the midbrain of PD rats established functional synaptic connections with the host midbrain DA system, thereby repairing the damaged neural circuitry. Piao et al. (2021) induced the differentiation of human embryonic stem cells (hESCs) into midbrain DA neurons and transplanted them into the brains of PD model mice; the results showed that the transplantation of these cells improved motor function, and hematological and biochemical analyses showed that the labeled transplanted cells neither migrated outside the brain nor formed tumors, providing a solid foundation for future clinical trials (Piao et al., 2021). However, attention should be paid when translating this transplantation technique into clinical practice. First, the safety and efficacy of clinical-grade hESC-derived dopaminergic neurons in non-human primate models need to be thoroughly evaluated. Wang et al. (2018) observed functional recovery after transplanting hESC-derived dopaminergic neurons into the brains of PD model monkeys. hESC cell lines that can be clinically applied have been successfully established, but issues related to immune rejection and ethical concerns have limited the large-scale use of these cells (Thomson et al., 1998; Nolbrant et al., 2017; Piao et al., 2021). Researchers have used somatic cell nuclear transfer (SCNT) to produce human parthenogenetic ESCs from unfertilized oocyte seeding cells for the treatment of PD, overcoming the ethical issues associated with ESCs. However, neural progenitor cells derived from ESCs are vulnerable to contamination, making it difficult to obtain an abundance of pure neural progenitor cells, which has always been a major challenge (Wang et al., 2018).

#### 4.3.2 Application of iPSCs in the clinical treatment of PD

Induced pluripotent stem cells, similar to ESCs, are pluripotent cells that are reprogrammed from somatic cells. Takahashi and Yamanaka (2006) first reprogrammed human fibroblasts into iPSCs by overexpressing four transcription factors (Oct3/4, Sox2, Klf4, and c-Myc). As iPSC technology has continuously advanced and been translated for clinical use, the number of transcription factors needed to reprogram cells into iPSCs has gradually decreased to two or even one, and some researchers have successfully produced iPSCs using recombinant proteins or even small molecules (Okita et al., 2011; Takahashi and Yamanaka, 2016; Guan et al., 2022; Liuyang et al., 2023). iPSCs are similar to ESCs in terms of gene expression profiles as well as their proliferation and differentiation capabilities. Compared to ESCs, iPSCs possess the advantages of being able to be implanted autologously due to being derived from patient-specific somatic cells, which may partially avoid ethical issues and immune rejection. However, iPSCs exhibit lower efficiency for neuronal differentiation and genetic instability after the reprogramming process, which might lead to 20q11 amplification, a genome abnormality commonly seen in cancer cells (Nguyen et al., 2018), and TP53 missense mutations (Merkle et al., 2017). Therefore, strict genomic screening of undifferentiated iPSCs after reprogramming is urgently needed to guarantee the safety of seeding cells (Parmar et al., 2020). The byproducts of 5-hydroxytryptamine (5-HT) neurons may cause gastrointestinal dysfunction (GID) concomitant with DA precursor neuron differentiation from iPSCs. To avoid this, Kikuchi et al. (2017) sorted DA precursor neurons using the floor plate marker CORIN and removed 5-HT neurons to obtain high-purity DA precursor neurons, which were then transplanted into the striatum of PD monkeys; the researchers found that the transplanted cells were able to survive in the long term (2 years) without forming tumors or causing immune rejection and that they could alleviate PD-related motor deficits and extend dense neurites into the host striatum regardless of whether the donors were patients or healthy individuals (Kikuchi et al., 2017). Cyranoski (2018) stimulated human induced pluripotent stem cells (hiPSCs) stored in the Kyoto University cell bank Takayuki Kikuchi to differentiate into DA precursor cells and transplanted the dopaminergic neuron precursor cells into the brain of a 50-year-old PD patient in the world’s first clinical trial of iPSC therapy for PD; there were no adverse reactions such as cerebral hemorrhage within 1 month after surgery [JPRN-UMIN00003356]. Schweitzer et al. (2020) injected DA progenitor cells derived from iPSCs into the striatum of an 80-year-old PD patient [FDA17145], and PET imaging showed that the transplanted cells survived well and improved motor function in the patient. iPSCs have been broadly applied in the clinical treatment of PD, but despite the rapid development of iPSC technology in recent years, there are still many issues to be overcome before they can be applied broadly in the clinic. First, the molecular mechanism of somatic cell reprogramming technology is not fully understood. Second, cells from the patient themselves may carry the risk of developing PD pathology (Goldman et al., 2019). Third, even when allogeneic iPSC-induced dopamine precursor cells are used, α-syn may diffuse from host cells to the seeding cells, causing the death of the transplanted cells (Hoban et al., 2020). Fourth, the efficiency and stability of directed differentiation *in vitro* still need to be improved.

#### 4.3.3 Clinical application of multipotent stem cells in the treatment of PD

Multipotent stem cells are undifferentiated cells with a self-renewal ability that can differentiate into cells of multiple lineages, including hematopoietic stem cells (HSCs), endothelial progenitor cells (EPCs), and MSCs (Bak et al., 2018; Staff et al., 2019; Liang et al., 2021). MSCs are easily obtained and have a low risk of immune rejection and good immunomodulatory and powerful paracrine effects, making them promising alternative seeding cells for PD treatment (Andrzejewska et al., 2021). Among the varieties of MSCs, bone marrow MSCs (BM-MSCs) were studied first and most widely, followed by adipose-derived MSCs (AD-MSCs) and umbilical cord-derived MSCs (UC-MSCs). Their functional mechanisms include the replacement of dopaminergic neurons, immune regulation, inhibition of apoptosis and inflammation, and neuroprotection (Chen et al., 2020; Heris et al., 2022). MSCs have remarkable potential in the treatment of PD, as they not only differentiate into dopaminergic neurons but also secrete a wide range of molecules, including exosomes containing miRNAs (Staff et al., 2019), which participate in intercellular signaling and crosstalk with host glial cells (Liu et al., 2022; Bhattacharyya et al., 2023). The MSC secretome plays a crucial role in regulating axonal growth and suppressing cell apoptosis. Moreover, MSC-secreted components can traverse the BBB and evade clearance by the reticuloendothelial system (Harrell et al., 2019; d’Angelo et al., 2020). To date, extensive efforts have been made to isolate secreted molecules from MSC culture medium to provide a foundation for the clinical application of the MSC secretome in the treatment of PD. Research has found that overexpression of the secretory component circSV2b prevents dopaminergic neuron loss, maintains nigrostriatal function and attenuates oxidative stress injury in an MPTP-induced PD mouse model (Cheng et al., 2022). Additionally, MSC-secreted miRNAs such as miR-133b and miR-17-92 have been shown to promote dendritic growth and regulate neurogenesis. Consequently, the MSC secretome is considered a promising therapeutic tool for PD, offering a novel avenue for the development of multitargeted treatment strategies (Xin et al., 2012, 2017). Nevertheless, there are many challenges in the application of the MSC secretome in PD therapy, such as how to optimize the components of the MSC secretome by eliminating harmful cell metabolites while harvesting beneficial ones; overcoming these challenge will allow the development of effective and efficient techniques for secretome isolation. Furthermore, direct transplantation of MSCs into the brains of PD model animals is another option worth exploring. Studies have shown that MSCs directly transplanted into the striatum of PD rats can partially differentiate into dopaminergic neurons expressing TH, while some MSCs differentiate into glial cells. These *in situ*-differentiated DA neurons survive as long as several months and restore DA release, resulting in improved motor function in PD rats (Tang et al., 2022). However, factors in extracellular vesicles derived from MSCs may cause excessive activation of microglia, promoting neuroinflammation and resulting in neuronal death (Baglio et al., 2015). Another study also demonstrated that MSC-derived exosomes activate microglia in PD rats (Zhang et al., 2023). A strategy in which MSCs are first primed to differentiate into neuron progenitor cells *in vitro* to make them more easily commit to the neuronal fate and more easily controlled than regular MSCs may be developed. Hayashi et al. (2013) transplanted DA neurons transdifferentiated from MSCs into the striatal area of PD model monkeys, and PET scans showed an increase in dopamine transporter (DAT) levels after transplantation, leading to improved motor function. Recent research has confirmed the safety and efficacy of MSCs in cell therapy, but standardized cell preparation protocols and clinical treatment strategies are still urgently needed. AD-MSCs can be conveniently obtained through liposuction or fat removal surgery, indicating the feasibility of autologous transplantation, and they also have the advantages of low immunogenicity, immunomodulatory potential, high genetic stability and good plasticity. UC-MSCs are similar, in that they have strong proliferation ability, high plasticity, low immunogenicity, and immunomodulatory potential. BM-MSCs and AD-MSCs can both be used for autologous transplantation; however, the method for obtaining BM-MSCs is invasive, and BM-MSCs have lower proliferation and differentiation potentials and can more easily become senescent during *in vitro* expansion. Moreover, the proliferative ability of MSCs may be reduced in PD patients due to ageing, limiting their therapeutic effect. UC-MSCs may be an important source of autologous or allogeneic cells for treatment, but whether human leukocyte antigen (HLA) is expressed on the cell surface of MSCs is not known, and further research is required (Fričová et al., 2020). A large amount of evidence has revealed that MSCs do not express human leukocyte antigen-II (HLA-II) and cannot be recognized by immune cells (Chuang et al., 2009; Sun et al., 2015). However, it has not yet been determined whether MSCs express HLA-II after committing to a functional cell fate. This question needs to be addressed both *in vitro* and *in vivo*.

Furthermore, Hay’s study demonstrates that AdMSCs can migrate toward prion-infected brain homogenate and produce anti-inflammatory molecules. *In vitro*, AdMSCs co-cultured with prion-exposed glial cells led to a significant reduction in inflammatory cytokine mRNA and markers of reactive astrocytes and activated microglia. This suggests that AdMSCs play a role in diminishing glial inflammation and could reprogram glial cells toward a healthier state (Hay et al., 2022). In the context of Parkinson’s disease, where DA neuron loss is a key pathological feature, the idea of diminishing glial dysfunction, in addition to replenishing DA neurons, is an intriguing prospect. The dual approach of addressing both neuron loss and glial cell dysfunction could offer a more comprehensive therapeutic strategy. While the specific literature focusing on DA neurons in this context might be limited, the broader concept of glial cell reprogramming and its impact on neuroinflammation is a viable area for future research. This approach aligns well with the current understanding of Parkinson’s disease pathology, where both neuron loss and neuroinflammation play critical roles.

### 4.4 Cell transplantation methods

#### 4.4.1 Intraventricular transplantation

Direct injection of cells into the damaged area of the brain represents the most precise delivery route for stem cell therapy. However, adverse reactions such as motor dysfunction, syncope, seizures, and tumorigenicity may occur in patients.

#### 4.4.2 Intravenous administration

Unlike *in situ* injection into a specific brain area, intravenous infusion of stem cells does not require surgery, trauma or other invasive techniques. However, intravenous delivery does not allow for cell homing and *in situ* differentiation into tissue-specific functional cells because engraftment is often uncontrollable and mostly depends on chemotaxis toward a bioactive gradient; this may result in off-target effects and low therapeutic efficiency and sometimes lead to the formation of ectopic tissue or even tumors.

#### 4.4.3 Nasal administration

To date, intranasal administration has proven to be a promising route for delivering therapeutic agents to the CNS due to the ability of intranasally administered agents to bypass the BBB. The incidence of adverse events is reduced in nasal administration compared with intraventricular transplantation, but the stem cell seeding efficiency is not compromised (Li Y. et al., 2017; Bagheri-Mohammadi et al., 2019). However, it should be noted that abundant cells remain in the upper nasal cavity 1 h after intranasal administration, indicating a low efficiency of stem cell migration (Galeano et al., 2018). No conclusive data on the cell entry pathways, related factors and cell type fitness have been reported, and further exploration is needed.

### 4.5 Immune rejection

Immune rejection has always been one of the most important challenges related to cell transplantation, and it is closely correlated with the survival of transplanted cells. Incompatibility between transplanted stem cells and the host immune system can lead to transplant rejection. The mismatch of the Human Leukocyte Antigen (HLA) system is a key factor leading to immune rejection in stem cell transplantation, mismatched HLA molecules can be recognized by the recipient’s immune system, triggering a rejection response. On the other hand, stem cells may exhibit epigenetic abnormalities during differentiation, leading to abnormal expression of immunogenic proteins. This process may cause the immune system to continuously attack and destroy the introduced exogenous stem cells, thereby suppressing their therapeutic effects (Zhao et al., 2011; Jin et al., 2016; Hay et al., 2022). The immune rejection response in stem cell transplantation is associated with three different allogeneic recognition pathways: the direct pathway, the indirect pathway, and the semi-direct pathway (Petrus-Reurer et al., 2021). Although the brain possesses immune privilege, immune rejection cannot always be avoided when incompatible tissues are implanted. In addition, the implantation procedure itself may disrupt the BBB, impairing the brain’s immune privilege and possibly allowing the entry of circulating immune cells into the brain (Tao et al., 2021). In early studies, immunosuppressive drugs were temporarily administered to prevent transplant rejection and improve the ratio of surviving neurons, but the optimal drug type, dosage and timing of use still need to be determined. Avoiding the long-term administration of expensive immunosuppressive drugs, which may be achieved through autologous stem cell-derived DA neuron transplantation, is necessary for the clinical treatment of PD (Madrid et al., 2021).

#### 4.5.1 Autologous cell transplantation for PD treatment

Autologous cell transplantation allows good immune compatibility, as patient tissue-derived iPSCs can avoid immune rejection after being differentiated into target cells before transplantation. Hallett et al. (2015) first demonstrated the feasibility of treating PD with autologous iPSC-derived DA neurons in non-human primate PD models. In crab-eating macaques with PD, unilateral implantation of iPSC-DA neurons was found to improve motor function without inducing an immune reaction around the iPSC grafts, as indicated by the lack of Iba1 expression in the host striatum, avoiding the need for immunosuppressants (Hallett et al., 2015). Tao et al. (2021) established an iPSC line from a PD primate model and transplanted cells differentiated from the iPSCs into PD model primates; this treatment strategy led to the restoration of fine motor skills (FMSs) (Gash et al., 1999). Resident immune cells, i.e., microglia (CD68) and astrocytes (GFAP), were not activated, and circulating CD3- and CD45-positive immune cells were nearly absent in the autologous grafts (Tao et al., 2021). Even for transplanted cells completely derived from the patient themselves, there is still a risk of rejection. However, autologous iPSCs can trigger immune reactions under certain conditions, as Zhao et al. (2011) found that autologous iPSC-derived DA neuron grafts triggered strong immune rejection in the mouse brain; the mechanism underlying this immune rejection has not yet been fully elucidated, but it may involve the introduction of mutations during iPSC preparation and expansion. Although autologous transplantation of iPSC-differentiated neurons has the advantage of a lower risk of immune rejection, allogeneic transplantation has the advantages of a shorter preparation time and the absence of PD-related gene mutations. In addition, MSCs, especially from autologously available adipose/fat or bone marrow, may also provide good alternatives for autologous cell transplantation. Hayashi et al. (2013) isolated MSCs from the bone marrow of crab-eating macaques with PD and induced them to differentiate into dopaminergic neurons *in vitro*. The differentiated cells were then injected into the striatum of PD model monkeys. According to the clinical rating scores (CRSs) and the reaching task, the monkeys showed an improvement in motor behavior. PET scans revealed the presence of C-labeled 2-β-carbomethoxy-3-β-(4-fluorophenyl)tropane (C-CFT) in the striatum, with no evidence of immunological reactions (Hayashi et al., 2013). Subsequently, Venkataramana et al. (2010) transplanted autologous BM-MSCs into the subventricular zone of seven PD patients in 2013. The patients’ motor symptoms were effectively ameliorated, as evaluated by the Unified-Parkinson Disease Rating Scale (UPDRS) score. The treatment was stable in three of the patients 3 years after surgery (Venkataramana et al., 2010). Zhou et al. (2013) transplanted autologous AD-MSCs extracted from rhesus macaques with PD into their brains. The AD-MSCs survived well and alleviated PD symptoms during a 4-month monitoring period (Zhou et al., 2013). Clinical trials of AD-MSCs for PD treatment are still ongoing, although no conclusive results have been reported yet. Conclusions that MSC transplantation is safe but has limited efficacy in animals can be tentatively drawn, but there are two important considerations. First, MSCs should be distinguished according to their origin, such as bone marrow, umbilical cord and adipose tissue, despite the fact that they are the same cell type. Second, the multidifferentiation potential of MSCs should be tightly controlled, and inducing the differentiation of MSCs into dopaminergic neurons before transplantation for PD treatment may be necessary to improve their therapeutic effects.

#### 4.5.2 Allogeneic transplantation for PD treatment

Preparing cells for allogeneic transplantation requires less time than autologous iPSC preparation and is cheaper in some respects, but allogeneic transplantation requires immunosuppression for a relatively long time. Screening for HLA-matched donor cells may reduce immune reactions. Previously, two strategies were developed to obtain HLA-matched donor cells: one involves screening natural HLA-matched donors, and the other involves disrupting both mismatched HLA-A and HLA-B alleles while retaining HLA-C, thus suppressing the natural killer (NK) cell response while maintaining antigen presentation (Morizane et al., 2017; Xu et al., 2019). Morizane et al. (2017) injected DA precursor cells derived from allogeneic monkey iPSCs into the putamen of other PD monkeys. HLA- A-, HLA- B-, and HLA-C-matched donor cells were sorted for transplantation; flow cytometry revealed low expression of major histocompatibility complex (MHC) in the grafts, and the transplanted allogeneic cells caused mild inflammation in the brain due to weak activation of microglia (Iba-1) and infiltration of leukocytes (CD45/CD3). However, staining with haematoxylin and eosin (H&E) and immunostaining indicated good survival of the transplanted cells, providing a solid foundation for later clinical trials (Morizane et al., 2013, 2017). In 2018, in a groundbreaking clinical trial, DA neuron precursor cells derived from allogeneic hiPSCs were transplanted into the putamen of PD patients and improved motor function (Schweitzer et al., 2020).

Notably, transplantation of iPSC-derived cells does not completely eliminate the risk of immune rejection (Hallett et al., 2014) The GForce-PD group advocates for short-term immunosuppression by at least one immunosuppressive agent, such as tacrolimus (FK506) or cyclosporine A (CyA), in clinical trials involving iPSC transplantation. In addition, triple immunosuppression for 1 year can lead to a higher cell survival ratio than administration of CyA for only 2 months post-transplantation (Barker et al., 2017; Kikuchi et al., 2017). To date, editing technologies, such as CRISPR/Cas9, have been used to edit mismatched HLA in iPSC cells to construct HLA-matched iPSC libraries (Deuse et al., 2019). However, this may not be possible since iPSCs maintained *in vitro* for a long period of time are too fragile to undergo additional gene editing via CRISPR/Cas9.

### 4.6 Can transplanted cells integrate into host neural circuits?

Cell therapy for PD must achieve not only neuronal replacement but also functional reinnervation of affected brain areas; this involves the integration of transplanted seeding cells into the host’s neural circuitry (Li et al., 2016). The implanted cells release DA in a precisely controlled manner rather than acting as DA pumps. The ability of transplanted cells to project axons to communicate with downstream neurons and receive inputs from upstream host neurons are key to the reconstruction of neural circuits, but related research is limited.

#### 4.6.1 Transplanted cells establish connections with host presynaptic cells

In early studies, synaptic connections between transplanted cells and neurons in the striatum were observed through electromicroscopy. In recent years, technologies such as optogenetics, immunostaining, and PET have revealed that transplanted cells can restore DA transmission and regulate the function of the host striatum in PD model animals (Grealish et al., 2014; Adler et al., 2019). Adler et al. (2020) used a rabies virus tracing system and demonstrated that DA precursor cells implanted into PD mice could project axons to the dorsolateral striatum, with these projections gradually dominating the host striatum over several months. Over time after surgery, the transplanted cells projected their axons to more distant areas in the forebrain, including the ventrolateral striatum, nucleus accumbens (NAc), and medial prefrontal cortex (PFC) (Cardoso et al., 2018; Adler et al., 2020). These findings confirm the capacity of the transplanted cells to project axons in the brain and promote the clinical application of cell therapy for PD patients.

For successful and functional integration into the host neural circuit, it is also necessary for seeding cells to receive presynaptic inputs from the host. In recent years, virus tracing technology combined with electrophysiology has revealed that several weeks after surgery, which coincides with the time when normal behavior is restored, cells transplanted into PD model animals receive projections from the host cortex and striatum (Cardoso et al., 2018; Linaro et al., 2019). Xiong et al. (2021) implanted DA precursor cells into the brains of PD mice and detected small amounts of spontaneous excitatory postsynaptic currents (sEPSCs) and spontaneous inhibitory postsynaptic currents (sIPSCs) in the transplanted cells through electrophysiology. Over time, the discharge frequency of sEPSCs and sIPSCs increased significantly, further supporting the idea that transplanted cells receive presynaptic inputs. However, it remains to be explored whether these inputs are crucial and necessary for the recovery of behavioral function in patients (Xiong et al., 2021). Regarding the long-term fate of transplanted cells, Li et al. (2008) reported that transplanted foetal mesencephalic dopaminergic neurons developed α-syn-positive LBs in two PD subjects after 11–16 years of survival. The recipients also reported symptomatic relief at these time points (Li et al., 2008). This evidence may provide insights into the crosstalk between the host and transplanted seeding cells.

#### 4.6.2 Optimizing the survival of transplanted cells

The host brain rejects transplanted cells after transplantation due to immune and inflammatory responses caused by mechanical damage that occurs during cell transplantation (Barker and Widner, 2004). This hinders the differentiation, maturation, and function of the transplanted cells (Ideguchi et al., 2008). The unfavorable environment of the brain may be corrected by transplanting cocultured glial cells and neurons. Song et al. (2018) cocultured astrocytes and mDA neurons to improve mDA neuron differentiation and maturation. The researchers then implanted the cocultured cells into the midbrain of PD rats, and the rats in the cocultured cell transplantation group exhibited more marked behavioral recovery than those in the mDA transplantation group (Song et al., 2018). This was related to the anti-inflammatory, antioxidant, and neurotrophic support provided by astrocytes (Han et al., 2021). This provides an inspiration for future research on whether the incidence of surgical injuries can be decreased and the survival rate of transplanted cells can be increased by transplanting cocultured mDA neurons.

Overall, these studies indicate that transplanted cells can extend axons, project these axons to relevant target areas, receive presynaptic inputs from host neurons, and functionally integrate into the host circuits, providing a theoretical basis for the use of stem cell replacement therapy for the treatment of PD. However, some complicated issues, including how transplanted cells sense inputs and are guided by the host microenvironment created by complex bioactive chemotaxis gradients, remain. Importantly, neural circuits in humans are much more sophisticated than those in mice. Future studies on non-human primates are urgently needed to evaluate whether implanted stem cells can integrate into host neural circuits and rescue PD-related functional impairments, with a much stronger focus on the long-term interaction between the host and the seeding cells.

## 5 Applications of pluripotent stem cells in PD modeling

Parkinson’s disease pathogenesis is complex, and to date, nearly one hundred PD-related genetic loci have been identified in genome-wide association studies (GWASs). However, the aetiology of PD remains a mystery due to the hundreds of single-nucleotide polymorphisms (SNPs) for each gene. Compared with animal models, *in vitro* hPSC models harboring specific PD-related gene mutations may better recapitulate PD pathology (Blauwendraat et al., 2019; Foo et al., 2020). In recent years, gene editing of hiPSCs has been successfully applied to model PD models *in vitro* (Ramos et al., 2021). Gene editing tools can be used to construct cells with genomic, epigenomic, and transcriptomic alterations, which represent powerful tools for studying PD pathogenesis (Hu et al., 2020; Kampmann, 2020). CRISPR/Cas9, which can achieve stable gene editing, has been used to validate PD-associated loci and even identify novel loci corresponding to subtle *in vitro* phenotypes (Hockemeyer and Jaenisch, 2016).

### 5.1 2D models of PD

Two-dimensional (2D) modeling based on stem cells is a valuable tool for simulating the progression of PD in humans under physiological conditions, allowing for the identification of cellular and molecular abnormalities that occur during PD development. This approach holds great promise for the identification of novel therapeutic targets in PD. The aggregation of α-syn, which encoded by *SNCA*, is a pathological hallmark of most cases of PD. iPSCs derived from patients with *SNCA* mutations exhibit intracellular aggregation of α-syn, leading to impaired anterograde axonal transport of mitochondria due to loss of nuclear factor erythroid-2 related factor 2 (Nrf2) transcriptional activity. ATP production is reduced in iPSCs harboring *SNCA* mutations, leading to energy deficiency for axonal transport and ultimately neuronal death (Prots et al., 2018; Czaniecki et al., 2019). Removal of both *SNCA* alleles results in reduced α-syn aggregation (Gandelman et al., 2021). Another study reported that treatment with the AKT modulator A-443654 can reduce the levels of α-syn and LBs in cultured iPSCs derived from patients with the 3X-*SNCA* mutation (Healy et al., 2008). *LRRK2* mutation is another major cause of PD. The G2019S point mutation in LRRK2 results in overactivity of LRRK2 kinase, which causes familial PD (Joyner et al., 2000; Orenstein et al., 2013). Companion-mediated autophagy (CMA) activation increases CMA lysosomal receptor levels in DA neurons derived from *LRRK2*-G2019S mutant iPSCs. CMA overactivation leads to elevated levels of α-syn. The *LRRK2* G2019S mutation also increases the expression level of monoamine oxidase-B (MAOB), exacerbating oxidative stress in cultured iPSCs and resulting in increased neuronal apoptosis (Heidari et al., 2022). Although many PD phenotypes at the genetic, epigenetic, transcriptomic and metabonomic levels can be mimicked in a 2D culture system, other essential phenotypes, such as loss of neuron-specific projections and cell communication, especially among microglia and DA neurons, cannot be modeled in a 2D culture system.

### 5.2 Application of 3D brain organoids for PD research

In most current PD research, DA neurons are cultured under 2D conditions. However, PD involves not only the loss of dopaminergic neurons in the substantia nigra but also the formation of α-syn-positive aggregates, which disrupt the function of other types of neurons and affect the brain’s microenvironment, including by inducing abnormal activation of glial cells, which may disrupt the normal function of DA neurons. The 2D DA neuron cultures used to simulate PD pathology lack the complex interactions among cells and cannot capture the complexity of the physiological microenvironment of the human brain. These requirements are crucial for modeling PD progression and identifying effective drugs with low toxicity (Yeap et al., 2023). Three-dimensional (3D) brain organoids are formed by the aggregation of both neuronal cells and non-neuronal supporting cells and therefore mimic specific brain regions. To construct human midbrain organoids (hMOs), SB, a suppressor of the Activin/TGF-β signaling pathway; the bone morphogenetic protein (BMP) inhibitor DMH1; and the glycogen synthase kinase 3β (GSK-3β) inhibitor CHIRlai1 were added to a suspended culture system (Tieng et al., 2014; Jo et al., 2016). Initially, upon induction of DA neuron differentiation, markers of progenitor cells, such as FOXA2, OTX2, CORIN, and LLIM homeobox transcription factor 1 alpha (LMX1), are expressed, after which markers of midbrain neurons, such as nuclear receptor related 1 (NURR1), TH, mammalian achaete-scute complex homologue-1 (MASH1), and engrailed homeobox 1 (EN1) are expressed. Finally, markers of mature DA neurons, such as G-protein-regulated inward-rectifier potassium channel 2 (GIRK2), are expressed, after which ventrolateral midbrain dopaminergic neurons and glial cells develop. The cellular composition, transcriptional features, membrane properties, and electrophysiological discharge patterns of hMOs are similar to those of cells in the human brain (Guan et al., 2022; Liuyang et al., 2023). Neurons in hMOs can generate coordinated electrical waves similar to those in the human brain, indicating a functional similarity between hMOs and the human brain (Smits et al., 2019; Nickels et al., 2020). 3D brain organoids can be used to elucidate the role of each cell subtype in the progression of PD and explore the pathogenesis of PD-related gene mutations, which may compensate for the limitations of 2D cell models and make clinical translation of stem cell transplantation possible (Fiore et al., 2022). In hMO with the 3X-*SNCA* mutation, the level of α-syn aggregates increases over time (Mohamed et al., 2021). Ha et al. (2020) developed a rapid method to generate midbrain-like structures (simBOs) using Shh and FGF8. Compared to controls, simBOs derived from PD patients with the *LRRK2* G2019S mutation exhibited increased LRRK2 activity, abnormal autophagy, and a decreased number of DA neurons, which were alleviated by the LRRK2 inhibitor PFE-360 (Ha et al., 2020). Brain organoids have broad application prospects, and their use in modeling PD pathogenesis and drug screening may lay the foundation for the development of future clinical treatments of PD. However, there are still some technical challenges. Genome editing during hiPSC differentiation may lead to off-target effects or transgene silencing (Zhang et al., 2017). In addition, during long-term culture or gene transfection, the genome of hiPSCs may become fragile and unstable, and genomic aberrations may occur occasionally (Martin et al., 2019; Sidhaye and Knoblich, 2021).

## 6 *In vivo* reprogramming of astrocytes or glia into neurons

**FIGURE 3:**
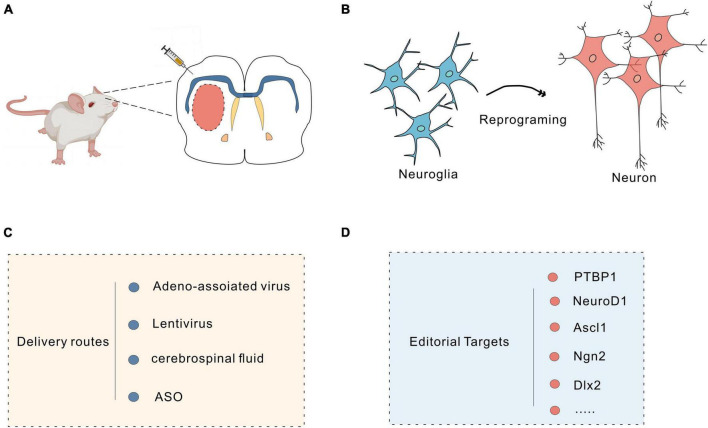
Neuronal reprogramming strategy. **(A)** Injection of vectors into the brain using a microinjector. **(B)** Schematic of the protocol used to reprogram glial cells into neurons. **(C)** Commonly used delivery routes for *in vivo* reprogramming. **(D)** Targets for reprogramming.

In mammals, neuronal loss due to traumatic brain injury or neurodegeneration is irreversible. Through *in vivo* cell reprogramming, cells can be transformed *in situ* to compensate for cell loss (Torper et al., 2013; Hockemeyer and Jaenisch, 2016); this method overcomes many of the difficulties faced with direct cell transplantation, such as immune rejection (Heinrich et al., 2015). Furthermore, the tissue environment promotes the functional maturation and timely integration of reprogrammed cells *in vivo* (Heinrich et al., 2015; Srivastava and DeWitt, 2016). Astrocytes are abundant in the brain, can proliferate massively in response to injury and are highly plastic (Yu et al., 2020). They are good targets for *in vivo* reprogramming of cells (Liddelow and Barres, 2017). Previous studies have demonstrated that fibroblasts can be efficiently transformed into functional neurons through *in vitro* chemical reprogramming (Li et al., 2015; Zhang et al., 2015; Li X. et al., 2017). Through advances in gene editing combined with biochemical techniques, researchers can reprogram target cells into pluripotent stem cells *in vivo*, and viruses, small molecules, and the CRISPR system can all be used as *in vivo* reprogramming tools (Figure 3).

### 6.1 Editing targets with viruses

Viruses, including retroviruses, lentiviruses and adeno-associated viruses (AAVs), can efficiently deliver specific promoters and transcription factors through targeted viral transduction to reprogram cells *in vivo* (Nectow and Nestler, 2020). PTBP1 (a factor associated with RNA) induces rapid and efficient astrocyte transformation in several regions of the CNS. Qian et al. (2020) reprogrammed astrocytes into functional dopaminergic neurons *in vivo* by knocking down the astrocyte-specific RNA-binding protein PTB with AAV-shPTB. Increased DA levels in the striatum of PD mice were observed using high-performance liquid chromatography (HPLC). Patch-clamp recordings revealed typical voltage-dependent currents and spontaneous postsynaptic currents in these transformed neurons, with sodium and potassium channel levels similar to those observed in endogenous DA neurons. These reprogrammed DA neurons were shown to integrate into the nigrostriatal dopamine circuit and to reconstruct damaged neural circuits and reverse motor deficits (Qian et al., 2020). In addition, Yang and colleagues effectively reprogrammed astrocytes into neurons by specifically knocking down PTBP1 in the striatum via CasRx. The results showed motor impairment was alleviated in PD model mice (Zhou et al., 2020). However, in other studies, knocking down or deleting PTBP1 failed to transform astrocytes into neurons, possibly due to viral leakage (Hoang et al., 2023; Yang et al., 2023). NeuroD1 is a helix-loop-helix proneural transcription factor that is essential for adult neurogenesis (Guo et al., 2014). Brulet et al. (2017) reported that astrocytes could be transformed into neurons in the mouse striatum by overexpressing NeuroD1. Subsequently, Leib et al. (2022) reported that NeuN-positive cells were observed after overexpression of NeuroD1 specifically near the site of virus injection in the mouse hippocampus and cerebellar cortex. Zheng et al. (2022) also reported that astrocytes could be reprogrammed into GABAergic neurons by overexpressing NeuroD1 in the hippocampus of epileptic rats; this reprogramming rescued pathological changes and decreased the seizure frequency. However, compared with Ptbp1 knockdown, NeuroD1 knockdown is much less efficient in converting astrocytes into neurons (Qian et al., 2020; Zhou et al., 2020) and is even less effective in the mouse brain and human brain. Therefore, studies on how the treatment efficiency of knockdown strategies can be improved, e.g., how to promote the maturation of offspring neurons and to distinguish the disease phenotype to minimize toxicity, are ongoing (Leib et al., 2022). In addition, several studies have shown that so-called “nascent neurons” generated in the presence of NeuroD1 are actually endogenous neurons, as many NeuroD1-mediated mCherry-positive neurons overlap with GFP-prelabelled endogenous neurons; this finding is controversial given that the NeuroD1 knockdown was found to induce the conversion of astrocytes into neurons (Wang et al., 2021). Some transcription factors may exert synergistic effects to induce cell reprogramming; the Gong and colleagues reprogrammed mouse striatal astrocytes into GABAergic neurons by manipulating the expression of two transcription factors, NeuroD1 and Dlx2, and achieved the highest cell conversion rate of 80%. Electrophysiological results indicated that the transformed neurons produced action potentials and integrated into the host neural circuit (Wu et al., 2020). Zhang et al. (2022) overexpressed a single transcription factor, DLX2, to induce the differentiation of ASCL1-expressing neural progenitor cells (NPCs) into astrocytes in the mouse striatum. Single-cell RNA sequencing (scRNA-seq) revealed that the DLX2-mediated reprogramming process is similar to the process by which NSCs are produced by neurogenesis. Immunohistochemistry showed that DLX2 and ASCL1 were colocalized in the early stages of reprogramming, suggesting that DLX2 induces ASCL1 expression in a cell-autonomous manner (Zhang et al., 2022). The proneural transcription factor ASCL1 is a master regulator of neurogenesis. Rao Z. et al. (2021) found that ASCL1 could induce the transformation of astrocytes into neurons. Among the targets of ASCL1, Klf10 is involved in neuritogenesis of iN cells in the early stage, Myt1 and Myt1l are critical for the electrophysiological maturation of iN cells, and Neurod4 and Chd7 are required for the efficient conversion of astrocytes into neurons (Rao Z. et al., 2021). Another study found that mutating six serine phosphorylation receptor sites in Ascl1 to Ascl1*^SA6^* could enhance the conversion of astrocytes into neurons in the mouse cerebral cortex (Ghazale et al., 2022). Giehrl-Schwab et al. (2022) developed the AAV-based intronic peptide split dcas9 activator system (AAV DCAS) by delivering Ascl1, Lmx1a NeuroD1, Lmx1a, Nr4a2 (ALN)/Ascl1, and miRNA218 (ALNe-218) in various combinations to efficiently reprogram striatal astrocytes into GABAergic neurons and integrate them into neural circuits, thereby improving voluntary motor function in a 6-OHDA-induced PD mouse model. Papadimitriou et al. (2023) injected a VSV-G pseudotyped lentivirus expressing miR-124-GFP into the mouse cortex and reprogrammed astrocytes into neurons by overexpressing miR-124; however, miR-124 overexpression alone was not sufficient to drive the full maturation of neurons, and the transformed cells were mainly immature neurons expressing TBR1 and NeuN. Whether transformed neurons can reestablish neural circuits and restore lost functions remains to be explored (Papadimitriou et al., 2023). Moreover, NG2 glial cells have been shown to play essential roles in neuronal repair. Guo et al. (2014) induced NG2 cells to reprogram into glutamatergic and GABAergic neurons by targeting NeuroD1. Tai et al. (2021) transformed NG2 glial cells in the mouse spinal cord into neurons by regulating SOX2 expression to achieve repair of the injured spinal cord. Microglial cells are the most regenerative glial cells within the CNS and are good targets for cell replacement and regeneration (Huang et al., 2018). In 2019, Kinichi Nakashima’s team from Japan induced microglia in the striatum of adult mice to transform into neurons by exogenous expression of NeuroD1 in mice via a lentivirus (Matsuda et al., 2019). The feasibility of microglia-neuron transdifferentiation is controversial, but evidence has consistently demonstrated that astrocytes and neurons can be reprogrammed into each other, as astrocytes and neurons are closely related and derived from the neuroectodermal lineage, both originating from radial glia. However, microglia develop from myeloid cells in the yolk sac, and their developmental lineage is distinct from that of neurons; therefore, the reprogramming of microglia into neurons is less feasible (Yu et al., 2020). Peng’s lab demonstrated in a series of experiments that NeuroD1 not only fails to induce microglia reprogramming *in vivo* but also causes microglial apoptosis. In addition, virus-mediated manipulation of PAX6, SOX2, ASCL1 and PTB1 expression failed to transform microglia into neurons. These results suggest that microglia-to-neuron reprogramming is unlikely to be achieved through the manipulation of a single gene (Rao Y. et al., 2021). Regarding the safety of cell reprogramming, viral transduction is currently the main strategy for reprogramming due to its high infection efficiency, the broad spectrum of cells that can be infected, and stable expression in offspring. However, retroviruses carry the risk of chromosomal instability and tumorigenicity, and non-integrated AAV delivery systems are highly toxic when delivered at high doses; therefore, the safety and efficacy of *in vivo* reprogramming via gene knockdown or overexpression should be fully considered to avoid potential off-target effects, mutagenesis, tumorigenesis and toxicity (Nectow and Nestler, 2020).

### 6.2 Editing targets using small-molecule mixtures

The use of small-molecule mixtures that target neural differentiation signaling pathways and transform astrocytes into neurons is a novel approach for cell reprogramming (Zhang et al., 2015; Ma et al., 2021). Ma et al. (2021) used DBcAMP, forskolin, ISX9, CHIR99021, I-BET151 and Y-27632 (DFICBY) to efficiently reprogram astrocytes into functional neurons *in vivo*. These chemically reprogrammed neurons exhibited electrophysiological properties and similarities to endogenous neurons. However, small molecule-treated glial cells can only be converted to glutamatergic and GABAergic neurons, and research on the targets for converting them to other neuronal subtypes is ongoing. Because of BBB, the drug dose required *in vivo* is usually higher than that required *in vitro* for the same effect, affecting clinical outcomes (Wang et al., 2022).

### 6.3 Editing targets with ASOs

Targeting PTBP1 with antisense oligonucleotides (ASOs), which can interfere with mRNA expression and regulate protein expression, can also convert astrocytes into neurons to reverse the disease phenotype. The Fu and colleagues injected PTB-ASO into the midbrain of transgenic mice. Immunofluorescence showed that a small fraction of tdTomato-labelled astrocytes were transformed into NeuN+ neurons. The electrophysiological results showed that the transformed neurons exhibited the physiological properties of endogenous neurons (Qian et al., 2020). Maimon et al. (2021) injected PTB-ASOs targeted specifically to astrocytes into the cerebrospinal fluid of mice to inhibit PTBP1 expression, and the immunofluorescence results showed a significant increase in tdTomato- and NeuN-labelled neurons in the hippocampus and cortex of mice. The electrophysiological results showed that the transformed neurons received both inhibitory and excitatory glutamate inputs and generated action potentials (Maimon et al., 2021). Currently, there are issues related to the dosage of viral vectors, and viral vectors present a risk of surgical injury; moreover, there are some challenges in delivering viruses to specific regions of the CNS. Injecting ASOs into the cerebrospinal fluid can effectively deliver them to the CNS in rodents and primates and reduce surgical risk (Smith et al., 2006). Currently, there are no other studies on whether PD progression can be reversed by the infusion of ASOs into the substantia nigra-striatal region. In the future, glial cells in the substantia nigra-striatal region could be transformed into DA cells by infusion of ASOs; this represents a new idea for clinical treatment.

Neuronal reprogramming is a promising strategy, and research has demonstrated the feasibility of reprogramming glial cells in the mouse brain. However, before moving to human trials, scientists need to conduct more animal studies to assess the survival, maturation, function and side effects of the reprogrammed neurons (Choi et al., 2020): (1) tracking transformed glial cells using spectral tracking techniques; (2) monitoring the transdifferentiation of glia into neurons in real time using two-photon microscopy; (3) using electrophysiology to analyze whether the new neurons integrate into the original neural circuits; and (4) performing extensive studies in primate models before conducting human experiments. Furthermore, whether there is a mechanism for maintaining cell fate should be determined. Differentiated cells can be induced to transform, so it is necessary to identify how cell identity can be protected to avoid the spontaneous transformation of example glial cells into neurons and damage to the nervous system. In conclusion, these studies offer new potential therapeutic options for PD and other neurodegenerative disorders.

## 7 Discussion

At present, the pathogenesis of PD remains to be fully elucidated. Moreover, traditional clinical treatments cannot fully reverse the progression of PD; therefore, the disease imposes a heavy burden on patients both emotionally and economically. With the development of cell therapy and gene editing technologies, pluripotent/multipotent stem cells may become key seeding cells for the clinical treatment of PD. Pluripotent/multipotent stem cells not only are novel tools for modeling PD but also hold great promise in restoring neuronal function through cell transplantation. In comparison to conventional treatment modalities, stem cells possess the remarkable abilities to self-renew and differentiate into various cell types. By transplanting the appropriate types of stem cells, dopaminergic neuron loss can be alleviated or these neurons can be replaced, resulting in amelioration of the motor or emotional symptoms of PD. Therefore, stem cells are ideal candidates for the clinical treatment of PD. Moreover, stem cells exert positive effects on damaged tissue by secreting bioactive molecules such as growth factors, neurotrophic factors, and inflammatory regulators. Through their powerful paracrine activity, stem cells have positive effects on the local microenvironment, promoting host tissue repair and thus functional recovery. Notably, acquiring stem cells from autologous sources such as skin or blood mitigates the risk of immune rejection and other possible incompatibilities. It can be predicted that the development of stem cell therapies tailored to individual patients will further enhance the clinical application of stem cells. DA neurons derived from ESCs, iPSCs and MSCs have entered clinical trials (NCT04414813, UMIN000048476, NCT05152394), bringing new hope for PD patients.

### 7.1 Ongoing challenges

There are still some challenges in clinical application (Barker et al., 2016). (1) Identifying long-term survival and functional integration of the transplanted cells will lead to ultimate therapeutic output, thus incurring broad concerns in the current cell therapy field. Various factors including microenvironment, cell dosage and donor-host compatibility would affect cell implantation. (2) Guaranteeing the safety of transplanted cells is crucial, because stem cell transplantation may lead to tumorigenesis and oncogenicity. Careful monitoring of both the transplanted stem cells and the host cells is needed. (3) Keeping the seeding cells in the targeted foci *in vivo* can be achieved by determining bioactive factors and their chemotaxis. (4) Determining how seeding cells gradually become heterogeneous, possibly due to microenvironment heterogeneity, is an important challenge. (5) Balancing the cost and cell quality when preparing and storing iPSCs and their derivative products is key. (6) Reducing immune rejection may become one of the foremost problems since successful cell survival *in vivo* may be the first step of effective cell therapy. (7) Identifying objective tools to evaluate seeding cell and host cell interaction outcomes, which determine long-term therapeutic effects, requires a large amount of clinical trial data to be analyzed and tracked.

### 7.2 Future research

Despite these challenges, PSCs/MSCs hold great promise for the treatment of PD. In 2023, the University Hospital of Skåne transplanted neural cells derived from ESCs into the brains of PD patients for the first time. It may take several years to validate their potential effects (LUND University). In the future, with a deeper understanding of stem cell biology, researchers can perform more precise cell engineering to produce more stable and safer transplanted cells that reduce potential risks, such as tumors, and to avoid immune rejection by altering surface markers on stem cells or developing new immunosuppressive drugs. Through the optimization of seeding cells, further studies on the genetic mechanism of PD, and improvements in cell delivery and real-time evaluation of seeding cell-host cell interactions, further success in clinical trials on PSC/MSC transplantation for PD can expected. In addition, more efficient cell transplantation routes may be developed in the future, such as the use of tissue engineering and organ culture techniques to build functional brain regions to better rebuild impaired function. Several international pharmaceutical companies are currently developing stem cell drugs for the treatment of PD, with Bayer (BAYGn.DE) subsidiary BlueRock reporting initial success using experimental stem cell therapies for the treatment of human PD and currently enrolling patients in a Phase II trial. In addition to the transplantation of exogenous stem cells to replace damaged dopaminergic neurons, *in vivo* reprogramming of astrocytes or glial cells is another option for PD treatment, although more research is needed to address issues associated with safety and long-term therapeutic efficacy.

Overall, the development of stem cell therapy for PD is still in the stage of continuous evolution and exploration. With continuous advancements in technology and a deeper understanding of PD pathogenesis, it is expected that PD can be completely cured in the future.

## Author contributions

YW played a significant role in the conception and design of the review and led the writing of the manuscript. XM co-led the conception and design of the review and contributed to the writing of the manuscript. W-YC participated in data collection and analysis. ZY assisted in data collection and analysis. KL helped shape the research, analysis, and manuscript. JW assisted in writing and editing the manuscript. TJ provided the critical feedback and guidance. FZ contributed to the overall direction and planning of the review and reviewed and edited the manuscript. K-HW oversaw the research direction and strategy and provided critical review and editing of the manuscript. CZ coordinated the research team and provided significant input into the design and structure of the review. YD supervised the project and reviewed and edited the manuscript. SG provided technical and fund support and participate in data interpretation and manuscript revision.
